# Differences in skin test reactions to official and defined antigens in guinea pigs exposed to non-tuberculous and tuberculous bacteria

**DOI:** 10.1038/s41598-023-30147-4

**Published:** 2023-02-20

**Authors:** Leire Fernández-Veiga, Miguel Fuertes, María V. Geijo, Bernat Pérez de Val, Enric Vidal, Lorraine Michelet, María Laura Boschiroli, Alberto Gómez-Buendía, Javier Bezos, Gareth J. Jones, Martin Vordermeier, Ramón A. Juste, Joseba M. Garrido, Iker A. Sevilla

**Affiliations:** 1 Departamento de Sanidad Animal, NEIKER-Instituto Vasco de Investigación y Desarrollo Agrario, Basque Research and Technology Alliance (BRTA), 48160 Derio, Bizkaia Spain; 2 IRTA, Programa de Sanitat Animal, Centre de Recerca en Sanitat Animal (CReSA), Campus de la Universitat Autònoma de Barcelona (UAB), 08193 Bellaterra, Catalonia Spain; 3 Unitat Mixta d’investigació IRTA-UAB en Sanitat Animal, CReSA, Campus de la UAB, 08193 Bellaterra, Catalonia Spain; 4 Laboratoire de Santé Animale, Unité Zoonoses Bactériennes, Agence Nationale de Sécurité Sanitaire de l’alimentation, de l’environnement et du Travail (ANSES), Université Paris-Est, 94701 Maisons-Alfort, France; 5 Centro de Vigilancia Sanitaria Veterinaria (VISAVET), Universidad Complutense de Madrid, 28040 Madrid, Spain; 6 Departamento de Sanidad Animal, Facultad de Veterinaria, Universidad Complutense de Madrid, 28040 Madrid, Spain; 7 Department of Bacteriology, Animal and Plant Health Agency (APHA), Surrey, KT15 3NB UK

**Keywords:** Immunology, Microbiology, Infectious-disease diagnostics

## Abstract

The single and comparative intradermal tuberculin tests (SITT and CITT) are official in vivo tests for bovine tuberculosis (TB) diagnosis using bovine and avian purified protein derivatives (PPD-B and PPD-A). Infection with bacteria other than *Mycobacterium tuberculosis* complex (MTC) can result in nonspecific reactions to these tests. We evaluated the performance of the skin test with PPDs and new defined antigens in the guinea pig model. A standard dose (SD) of *Rhodococcus equi*, *Nocardia* sp., *M*. *nonchromogenicum*, *M*. *monacense*, *M*. *intracellulare*, *M*. *avium* subsp. *paratuberculosis*, *M*. *avium* subsp. *avium*, *M*. *avium* subsp. *hominissuis*, *M*. *scrofulaceum*, *M. persicum*, *M*. *microti*, *M*. *caprae* and *M*. *bovis*, and a higher dose (HD) of *M*. *nonchromogenicum*, *M*. *monacense*, *M*. *intracellulare*, *M*. *avium* subsp. *paratuberculosis* were tested using PPD-B, PPD-A, P22, ESAT-6-CFP-10-Rv3615c peptide cocktail long (PCL) and fusion protein (FP). The SD of *R*. *equi*, *Nocardia* sp., *M*. *nonchromogenicum*, *M*. *monacense*, *M*. *intracellulare* and *M*. *avium* subsp. *paratuberculosis* did not cause any reactions. The HD of *M*. *nonchromogenicum*, *M*. *monacense*, *M*. *intracellulare*, and *M*. *avium* subsp. *paratuberculosis* and the SD of *M*. *avium* subsp. *hominissuis*, *M*. *scrofulaceum* and *M*. *persicum*, caused nonspecific reactions (SIT). A CITT interpretation would have considered *M*. *avium* complex and *M*. *scrofulaceum* groups negative, but not all individuals from *M*. *nonchromogenicum* HD, *M*. *monacense* HD and *M*. *persicum* SD groups. Only animals exposed to *M. bovis* and *M. caprae* reacted to PCL and FP. These results support the advantage of complementing or replacing PPD-B to improve specificity without losing sensitivity.

## Introduction

Animal tuberculosis (TB) is a globally spread zoonotic disease caused by species encompassed by the *M*. *tuberculosis* complex (MTC), most importantly by *M*. *bovis* and *M*. *caprae*. MTC includes other animal-adapted variants, namely *M. microti*, *M. pinnipedii*, *M. orygis*, *M. mungi*, *M. suricattae*, the Dassie bacillus and the chimpanzee bacillus^1^. It is a notifiable disease listed in the Terrestrial Animal Health Code (TAHC) of the World Organisation for Animal Health (WOAH). The disease has a great economic impact in the livestock industry and is a recognized public health problem due to its zoonotic nature^1^. One of the reasons for its economic impact lies in the huge costs derived from the implementation and application of strategies to control the disease. Apart from post-mortem inspection of animals at slaughterhouses in search of lesions compatible with TB, the tuberculin skin test (TST) is the standard diagnostic method for in vivo detection of infected individuals in TB eradication programs^2^. The European Communities Commission recognizes the single intradermal tuberculin test (SITT) and the comparative intradermal tuberculin test (CITT) as the official assays in the Member States, and the interferon-gamma (IFN-γ) assay as an alternative test that needs to be carried out in the laboratory (Commission delegated Regulation (EU) 2020/689 of 17 December 2019). In the SITT, the tuberculin known as purified protein derivative (PPD) from *M*. *bovis* (PPD-B) is injected intradermally and the skin-fold thickness is measured. When an animal is infected with or has been exposed to MTC bacteria, a delayed hypersensitivity reaction occurs causing an increase in the skin-fold thickness and/or clinical signs (diffuse or extensive edema, exudation, necrosis, pain or inflammation of the lymphatic ducts in that region or of the lymph nodes) at the PPD-B injection site. In the CITT both PPD-B and a PPD obtained from *M*. *avium* (PPD-A) are injected for comparison of the reactions. Sensitization with some non-tuberculous mycobacteria (NTM) or vaccination against TB or paratuberculosis, among other factors, have been pointed out as factors that can affect the specificity of the SITT^3,4^, leading to the slaughter of non MTC-infected animals and causing economic losses and great concern among farmers and authorities. The CITT can solve some of these issues by turning into negative some unspecific reactions to PPD-B. Its specificity can reach values of up to 99.98%^5^ but can miss some MTC-infected individuals reducing its sensitivity^2^. Antigenic reagents other than standard PPDs have been developed. Some have the capability to differentiate infected from Bacille Calmette-Guérin (BCG)–vaccinated animals (DIVA) because they are based on regions that are absent or not immunogenic in BCG vaccine strains (ESAT-6, CFP-10 and Rv3615c)^6^, like the defined antigens peptide cocktail long (PCL) and triple fusion protein (FP) used in previous studies^7,8^. A protein complex affinity-purified from the PPD-B called P22 has been developed as an alternative antigen in TB antibody based diagnosis^9^. This antigen has been recently used also in cellular response based tests^10^. Some NTM have been isolated from animals that reacted to PPDs^11^. However, few experimental studies on cross reactive immune responses induced by NTM exposure have truly demonstrated their ability to interfere with tuberculins in the skin test or the IFN-γ release assay^12,13^. Thus, the objective of this study was to evaluate the cross-reactivity of different NTM and other bacteria that were previously isolated from non-MTC-infected cattle reacting to skin testing or displaying TB-compatible lesions with official and new defined skin test antigens in guinea pigs exposed to these microorganisms.

## Results

### Skin test

**Figure 1:**
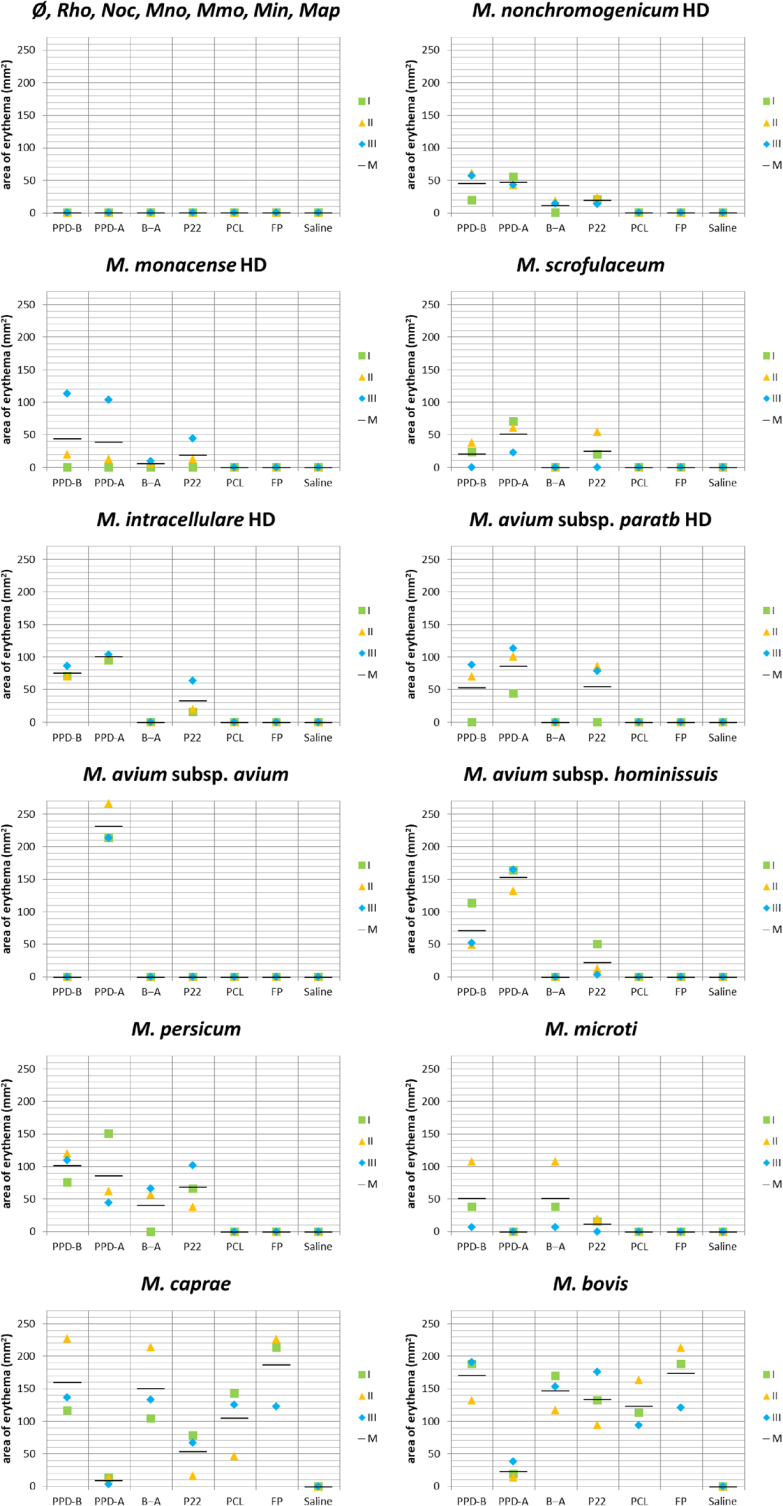
Reactivity according to inoculated organism and test antigen. Individual (I, II and III) and mean (M) erythema areas read 24 h after skin test antigens inoculation are plotted. The panel at the left on the top serves to represent the control group that was not inoculated any organisms (Ø), and the standard dose (SD) of *Rhodococcus equi* (*Rho*), *Nocardia* sp. (*Noc*), *M. nonchromogenicum* (*Mno*), *M. monacense* (*Mmo*), *M. intracellulare* (*Min*) and *M. avium* subsp. *paratuberculosis* (*Map*). The rest of results correspond to the SD of the organism indicated except for *M. nonchromogenicum*, *M. monacense*, *M. intracellulare* and *M. avium* subsp. *paratuberculosis* (*M. avium* subsp. *paratb*) that correspond to inoculations with the higher dose (HD). B‒A indicates the result of subtracting PPD-A result to PPD-B result (PPD-B‒PPD-A).

No animals, experimental units or data points were excluded from the study. Guinea pigs from control group (no sensitization) did not display any reaction to any of the skin test antigens used. The sizes of skin reactions (erythemas) seen after 24 h in response to intradermal inoculation of skin test antigens are shown in Fig. 1. No reaction was detected at the site where saline solution was inoculated in any of the groups. No large deviations from the mean were observed in each group. Most inoculated animals developed skin reactions to PPD-B and P22, except for non-mycobacterial agents and standard dose (SD) mycobacteria groups that were repeated (*M*. *nonchromogenicum*, *M*. *monacense*, *M*. *intracellulare* and *M*. *avium* subsp. *paratuberculosis*) using a higher sensitization dose (HD). These HD inoculations were performed because, in contrast with previous reports and findings (i.e. isolation of these NTM from reactor cattle not infected with MTC), no reactions were observed with the SD and testing a HD was deemed necessary. Testing a HD of non-mycobacterial agents or of the remaining NTM was ruled out because the hypothesis was not planned beforehand, there was limited availability of animal resources and biosafety level 3 animal facilities at the time the second experiment could be conducted and the potential information that could be gained was not considered sufficient to ask for a modification of the authorization for animal experimentation and outweigh the “reduce” principle of the three Rs. All MTC-sensitized groups other than *M. microti* group developed strong reactions to antigens PCL and FP, especially to FP, while no reaction was detectable in the remaining guinea pigs groups. The specific response of animals sensitized with *M*. *microti* to PPD-B and P22 was much lower than that displayed by the other two MTC organisms. Erythematous area size analysis of variance showed a significant effect of antigen (*p* < 0.0001), sensitizing organism (*p* < 0.0001) and time (*p* < 0.0001). Antigen by organism (*p* < 0.0001) and antigen by time (*p* = 0.0481) were significant interactions, but not organism by time (*p* = 0.8525) nor antigen by organism by time (*p* = 0.9935) (Table 1). The mean PPD-B erythema area in *M*. *bovis*-sensitized group significantly differed from the remaining groups, except for the *M*. *kansasii* complex (MKC) member *M. persicum* (*p* = 0.5581) (see Table 2). Using P22, *M*. *persicum* and *M*. *avium* subsp. *paratuberculosis* HD-sensitized groups were not significantly different from *M*. *bovis* in their erythema area size. Comparison of PPD-B response of *M*. *caprae*-sensitized guinea pigs with other groups yielded smaller but still significant differences, except with *M*. *persicum* (*p* = 0.8688), *M*. *avium* subsp. *hominissuis* (*p* = 0.0837) and *M*. *intracellulare* HD (*p* = 0.1415) groups. This was not the case for P22, for which no differences were found with any group displaying reactions. The highest mean reactivity of non-MTC sensitizations (*M. persicum* group) was lower than specific *M*. *bovis* or *M*. *caprae* mean reactivity towards PPD-B. However, the erythema area of one guinea pig from the group sensitized with *M*. *persicum* fell within the range of *M*. *caprae*-sensitized group. Trying to set a cutoff with P22 for differentiating between positive and negative individuals was more difficult because there were two sensitizations (*M*. *persicum* and *M*. *avium* subsp. *paratuberculosis* HD) that caused reactions even higher than the expected specific one with *M*. *caprae*. MTC-sensitized groups (except the *M. microti* group) developed strong reactions to antigens PCL and FP, especially to FP, while no reactions were detected in groups exposed to bacteria other than MTC. The specific response (PPD-B and P22) of animals sensitized with *M*. *microti* was much lower than that displayed by the other two MTC members.

**Table 1:** Analysis of variance for erythema area depending on the organism, the antigen and the reading time.

		Initial model (R^2^: 0.8832; *p* < 0.0001)			Final model (R^2^: 0.8546; *p* < 0.0001)		
Factor	Level	n	Mean	SD	n	Mean	SD
Organism	*M. bovis* SD	36	93.56	73.83	12	150.67	41.04
	*M. caprae* SD		85.82	81.24		126.68	69.01
	*M. microti* SD		8.91	24.65		15.74	31.41
	*M. persicum* SD		36.00	44.21		42.61	49.21
	*M. scrofulaceum* SD		8.03	18.68		11.33	18.70
	*M. avium* subsp. *hominisuis* SD		32.81	51.91		23.37	35.77
	*M. avium* subsp. *avium* SD		32.61	75.89		0.00	–
	*M. avium* subsp. *paratuberculosis* HD		26.22	37.69		26.97	40.05
	*M. intracellulare* HD		29.04	37.33		27.23	34.70
	*M. monacense* HD		12.29	30.14		15.78	33.42
	*M. nonchromogenicum* HD		14.07	19.15		15.97	21.84
	*Nocardia* sp. SD		0.00	–		–	–
	*Rhodococcus equi* SD		0.00	–		–	–
	None		0.00	–		–	–
Antigen	PPD-B	84	52.08	62.27	33	72.17	60.53
	PPD-A		47.65	61.30	–	–	–
	P22		25.52	38.31	33	40.08	43.19
	Fusion protein (FP)		24.07	62.13		32.89	73.29
	Peptide cocktail long (PCL)		14.69	39.37		20.80	47.61
	Saline solution		0.0	–	–	–	–
Time	24 h	252	31.57	56.17	132	41.48	27.83
	48 h		22.77	47.52	–		–

The initial model includes all effects and their interactions. The final model only retains sensitizing organism and antigen (except PPD-A, because the main goal was to assess the interference in SITT diagnosis) only at the levels that showed any reaction. It is indicated if it was a standard dose (SD) or a higher dose (HD).

*p* model probability of the effects not causing any difference in the mean of the dependent variable (erythema area), *n* number of observations for each of the levels under analysis, *sd* standard deviation.

**Table 2:** Statistical significance (*p* values) of the differences in skin test mean reactions to PPD-B and P22 observed in the NTM- (and *M. microti*)-sensitized guinea pig groups as compared to those seen in *M*. *bovis* and *M*. *caprae*-sensitized guinea pig groups.

	PPDb		P22	
	*M. bovis*	*M. caprae*	*M. bovis*	*M. caprae*
*M. microti* SD	0.0008	0.0046	0.0005	0.9988
*M. persicum* SD	0.5581	0.8688	0.6569	1
*M. scrofulaceum* SD	< 0.0001	< 0.0001	0.0042	1
*M. avium* subsp. *hominissuis* SD	0.0208	0.0837	0.0026	1
*M. avium* subsp. *avium* SD	< 0.0001	< 0.0001	< 0.0001	0.9427
*M. avium* subsp. *paratuberculosis* HD	0.0011	0.006	0.2359	1
*M. intracellulare* HD	0.039	0.1415	0.0148	1
*M. monacense* HD	0.0002	0.0014	0.0016	1
*M. nonchromogenicum* HD	0.0003	0.0017	0.0015	1

It is indicated if it was a standard dose (SD) or a higher dose (HD).

**Figure 2:**
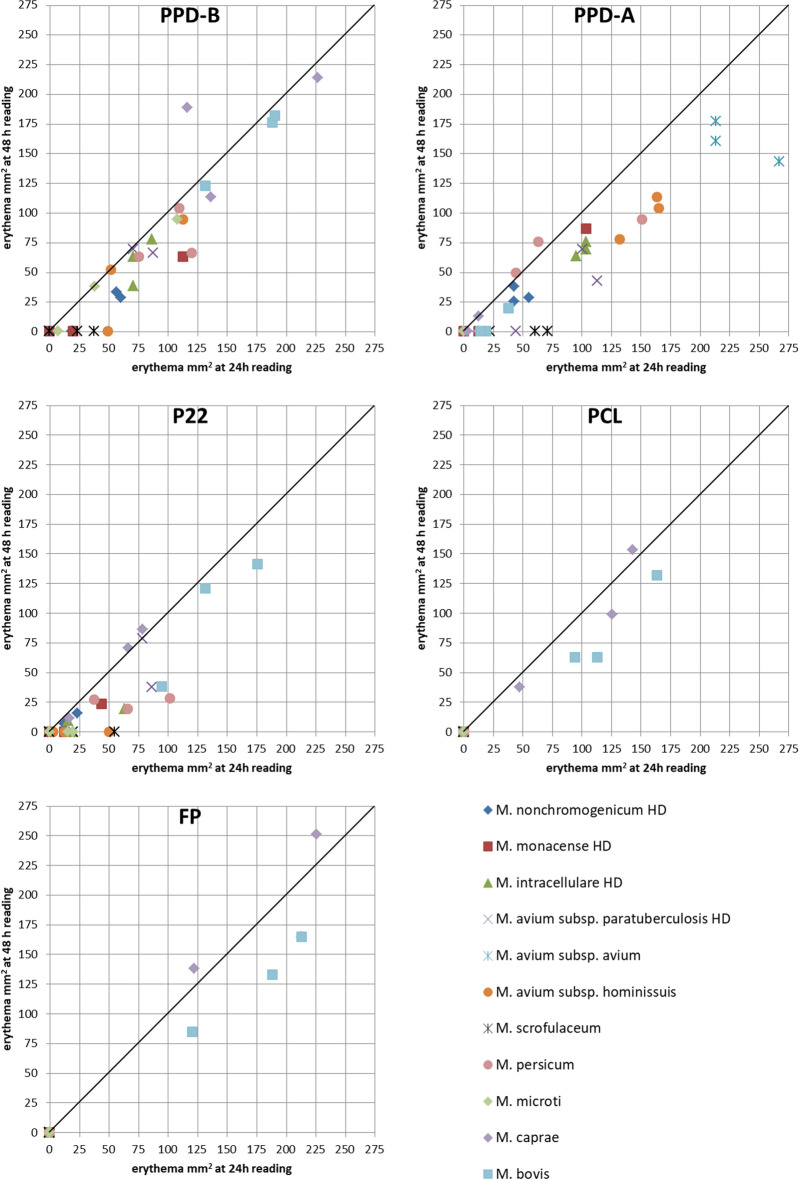
Effect of the time of reading (24 or 48 h) on the erythematous area according to inoculated organism and intradermal antigen. Results refer to inoculations with the standard dose (SD) except for *M. nonchromogenicum*, *M. monacense*, *M. intracellulare* and *M. avium* subsp. *paratuberculosis* that correspond to inoculations with the higher dose (HD).

Antigen mean reading at 24 h (31.57 mm^2^) significantly decreased by 28% at 48 h (22.77 mm^2^) (*p* < 0.0001) (Table 1). Both values were well correlated when the first reading was above 50 mm^2^, but most reactions below this value vanished by 48 h (Fig. 2).

### Post mortem analysis

Post-mortem results are summarized in Table 3. No macroscopic or microscopic lesions were detected among guinea pigs from *Nocardia* sp., *R*. *equi*, *M*. *nonchromogenicum* SD and HD, *M*. *monacense* SD and HD, *M*. *intracellulare* SD, *M*. *avium* subsp. *paratuberculosis* SD and *M*. *scrofulaceum* groups and, none of them yielded any isolate. Two *M*. *intracellulare* HD-sensitized individuals showed microscopic lesions compatible with mycobacterial infection that were not confirmed as being of mycobacterial origin by Ziehl–Neelsen staining (ZN) or culture. All three were culture negative. One guinea pig from the *M*. *avium* subsp. *paratuberculosis* HD group showed granulomatous lesions in liver and mesenteric LN that were not confirmed by ZN though the pool containing liver (pool 3) was culture positive with a single colony growing on a Herrold’s Egg Yolk Agar slant. Pool 1 containing the mesenteric LN of this animal was culture negative. In the *M*. *avium* subsp. *avium* group, two individuals were pool 3 culture positive and one of them had also lesions in muscle tissue at the inoculation site that was confirmed by culture. Similarly, the culture of pool 3 of two guinea pigs included in the *M*. *avium* subsp. *hominissuis* group produced isolates. One of them had granulomatous lesions in liver and spleen and, though ZN was negative, the isolation of *M*. *avium* subsp. *hominissuis* from pool 3 suggested that lesions could be due to this mycobacterium. As for the *M*. *persicum* group, gross and microscopic lesions were observed in one guinea pig (liver and mesenteric lymph nodes) and the bacterium was isolated from pools 1 and 3 in 2 individuals, from pool 2 in one animal and from the inoculation site also in one case. With regard to MTC, in the *M*. *microti* group lesions were only detected in the liver of one guinea pig but the bacterium was not isolated from any of these animals. In contrast, all guinea pigs belonging to *M*. *caprae* and *M*. *bovis* groups displayed lesions and were culture positive (Table 3).

**Table 3:** Culture and pathological post-mortem results of the 3 guinea pigs of each group.

Inoculated organism and dose	Culture positive (n/3)				Lesions location (n/3)
	Pool 1	Pool 2	Pool 3	Muscle^	
*Nocardia* sp. SD, *Rhodoccocus equi* SD, *M. nonchromogenicum* SD and HD, *M. monacense* SD and HD, *M. intracellulare* SD, *M. avium* subsp. *paratuberculosis* SD and *M. scrofulaceum* SD	0/3	0/3	0/3		0/3
*M. intracellulare* HD	0/3	0/3	0/3		Liver (2/3) (not confirmed by ZN or culture)
*M. avium* subsp. *paratuberculosis* HD	0/3	0/3	1/3		Mesenteric LN and liver (1/3) (not confirmed by ZN and not demonstrable by culture; but the pool containing liver (Pool 3) was culture-positive)
*M. avium* subsp. *avium* SD	0/3	0/3	2/3	1/3	Muscle (1/3)
*M. avium* subsp. *hominissuis* SD	0/3	0/3	2/3		Liver and spleen (1/3) (not confirmed by ZN and not demonstrable by culture; but the pool containing liver and spleen (Pool 3) was culture positive)
*M. persicum* SD	2/3	1/3	2/3	1/3	Mesenteric LN, liver and muscle (1/3)
*M. microti* SD	0/3	0/3	0/3		Liver (1/3)
*M. caprae* SD	0/3	3/3	3/3	2/3	Lung (1/3), tracheobronchial LN (3/3), liver (3/3), spleen (3/3), retropharyngeal LN (1/3), iliac LN (3/3), hepatic LN (3/3), prefemoral LN (2/3), popliteal LN (2/3) and muscle (2/3)
*M. bovis* SD	2/3	2/3	3/3	2/3	Liver (3/3), spleen (3/3), iliac LN (3/3), hepatic LN (3/3), prefemoral LN (2/3), inguinal LN (3/3), popliteal LN (2/3), and muscle (2/3)

Cultured pools include intestine and mesenteric LN (Pool 1), lungs and associated LN (Pool 2) and liver, spleen and retropharyngeal, parotid, cervical, prescapular, axillary, iliac, hepatic, prefemoral, inguinal and popliteal LNs (Pool 3).

*LN* lymph node, *SD* standard dose of bacteria, *HD* higher dose of bacteria.

^Muscle tissue from bacteria inoculation site (cultured only if macroscopic lesions present).

## Discussion

Exposure to or infection with some NTM or other bacteria have been considered as potential causes of nonspecific sensitization to PPD-B in the skin and other diagnostic tests of TB in cattle^3,13,14^. In spite of this, few controlled experiments have been carried out to demonstrate the implication of non-tuberculous organisms in the occurrence of cross-reactions with PPD-B and to evaluate the degree of interference of these nonspecific reactions in the diagnosis of TB. In addition, apart from some studies focused mainly on *M. kansasii* complex^15–17^, reports on the performance of antigens other than PPD (i.e. defined antigens) in tests based on cellular immune response and focused on non-MTC sensitizations are lacking. In this study we wanted to assess the degree of cross-reactions caused by sensitization (or infection) with some NTM strains and other non-mycobacterial microorganisms in response to skin test inoculation of standard PPDs and newer defined antigens using guinea pigs and isolates recovered from TB-negative cattle reacting to the skin test or with TB-compatible lesions.

Inoculation with the SD of *Rhodococcus equi*, *Nocardia* sp., *M. nonchromogenicum*, *M. monacense*, *M. intracellulare* and *M. avium* subsp. *paratuberculosis* did not cause any visible skin reactions. According to these results these microorganisms would not cause interferences in the skin test. However, in the light of previous research^11,13,14^ that associated the appearance of cross-reactive responses in TB diagnostic tests with the detection of these NTM in the animals displaying those responses, we decided to increase the amount of mycobacteria administered to test whether this was due to a dose effect. Due to the limitations mentioned before (in skin test results), we had to prioritize and performed HD inoculations with the specified NTM and discarded *Nocardia* sp. and *C. pseudotuberculosis*. The latter two microbes have been associated with interferences in the diagnosis of bovine TB^3,18^, but their interferences might be more important as confounders in abattoir inspection (causing TB-like lesions) than in triggering cross-reactive responses^18,19^. Inoculations with *M. nonchromogenicum*, *M. monacense*, *M. intracellulare* and *M. avium* subsp. *paratuberculosis* were repeated on new guinea pig groups with a HD and animals did develop visible reactions to PPDs and P22. This indicates that the response to diagnostic antigens is dependent on the dose of sensitizing bacteria (at least with respect to these species). Accordingly, nonspecific responses are expected to be greater the higher the sensitizing dose used is. Little is known on the dose of NTM needed to establish an infection or to induce a detectable reactive response in an animal under natural conditions. However, high amounts of NTM are found in soil or dust (up to 10^6^ CFU/g), different types of water (up to 10^4^ CFU/ml), biofilms (up to 10^6^ CFU/cm^2^), plants (> 10^6^ CFU/g), contaminated feed (e.g. feed stored in contact with domestic fowl), invertebrates, etc.^3,20–23^. Animals can get exposed to these sources by occasional or repeated contact through the respiratory or digestive tract mucosae or skin injuries. Unfortunately, there is a lack of information on the concentration of NTM harbored by infected animals; most studies dealing with NTM detection both in livestock and wildlife only report the prevalence or the number of culture positive individuals/tissues. Altogether, our results show that sensitization with *M. nonchromogenicum* HD, *M. monacense* HD, *M. intracellulare* HD, *M. avium* subsp. *paratuberculosis* HD, *M. avium* subsp. *hominissuis* SD, *M. scrofulaceum* SD and *M. persicum* SD could make animals react to the SITT. Most of these species have been previously pointed out as potential causes of nonspecific skin test reactions^3,13,14^. In contrast to previous research^3,14^, none of the animals from *M. avium* subsp. *avium* group displayed reactions to PPD-B or P22, despite their strong response to PPD-A. This isolate was obtained from a reactor cow confirmed as not infected with MTC. It could be that the standard dose was sufficient to produce top responses towards PPD-A but insufficient to cause visible responses to PPD-B. An alternative explanation for this would be that infection of guinea pigs with *M. avium* subsp. *avium* induced an immune response specific to targets in PPD-A that are not present (or under represented) in PPD-B and P22, at least in this experiment.

Interestingly, a comparative interpretation based on PPDs (CITT) would have deemed skin test negatives all individuals from *M*. *avium* complex and *M. scrofulaceum* groups as well as some from *M. nonchromogenicum* HD, *M. monacense* HD and *M. persicum* SD groups. Regardless of the specificity gain of this comparative interpretation, other research showed that sensitivity can be affected because animals co-infected with NTM (especially *M*. *avium* subspecies) and MTC can go undetected for having greater responses to PPD-A than to PPD-B^24–26^. In any case, since PPD-B erythema areas were greater than those seen for PPD-A, *M. nonchromogenicum* HD, *M. monacense* HD and, in particular, *M. persicum* could still have the potential to interfere also in the CITT. Another relevant point to be highlighted is that many of the animals belonging to NTM groups and reacting to the skin test did not show lesions nor yielded any isolates. The impossibility of confirming or ruling out that a reactor animal’s response is due to sensitizations with non-tuberculous organisms represents a setback for eradication programs, additional to the occurrence of some truly MTC-infected reactors for which tuberculous infection cannot be confirmed due to the low sensitivity of confirmatory tests^14^.

The results presented here show a high level of consistency and a relatively low variability that allowed obtaining statistical support to the differentiation of reactions. But the limited number of animals in each group precluded us from determining diagnostic cutoffs and thus, estimating the diagnostic performance parameters of antigens reliably. A general drop of erythema areas read at 24 h was observed at 48-h reading, with most reactions below the size of 50 mm^2^ disappearing by 48 h (Fig. 2). But some unspecific reactions to PPD-B still remained above 50 mm^2^ after 48 h (*M. monacense* HD, *M. intracellulare* HD, *M. avium* subsp. *paratuberculosis* HD, *M. avium* subsp. *hominissuis* SD and *M. persicum* SD). This and other considerations aside, we used 24-h readings comparisons following the recommendations of the WOAH for tuberculin potency testing in guinea pigs^27^. Mean PPD-B reactions were significantly greater in the group of *M. bovis* compared to the rest of groups except for that of *M. persicum* (*p* = 0.5581). Previous research showed that the skin-fold thickness increase in response to PPD-B was significantly smaller (*p* > 0.05) in MKC-challenged cattle than in *M. bovis*-challenged cattle^15^. This disagreement may be explained by the differences in challenge doses and bacterial strains used in each study. This was not the case for *M. caprae* group, with mean responses not significantly different not only in comparison with *M. persicum* but also with *M. avium* subsp. *hominissuis* or *M. intracellulare* HD groups. When it came to determine a cutoff to consider individuals as reactors or non-reactors, some of the animals sensitized with *M*. *persicum*, *M. avium* subsp. *hominissuis* and *M. monacense* HD showed reactions almost equal or even greater than those observed in one *M*. *caprae* guinea pig, which would have reduced the specificity or the sensitivity of PPD-B depending on the cutoff selected.

P22 has been proven to be useful and more specific than PPD-B in detecting the humoral response of infected animals^9,28^. However, its performance as a skin test reagent has not improved that of PPD-B in these experiments with guinea pigs. It did not improve specificity, with reactions not significantly different between *M*. *persicum*, *M*. *avium* subsp. *paratuberculosis* HD and *M. bovis* groups and between *M. caprae* and the rest of groups. It is striking that the difference between reaction sizes to PPD-B (larger) and P22 (smaller) is notably greater in the *M*. *caprae* group than in the *M*. *bovis* group. This may be because P22 is immunopurified from *M*. *bovis*-derived PPD-B and some of the antigens selected to compose this protein sub-complex may be less abundant, less immunogenic or down expressed in our *M. caprae* isolate. In spite of this, the mean erythema area of *M*. *caprae* group in response to PPD-B and P22 was greatly influenced by the guinea pig that had the largest reaction to PPD-B and the smallest one to P22 in its group. A field study on *M. caprae*-infected goats reported a sensitivity of 94% and 87% for SITT and P22 skin test, respectively^10^. But the mean and median SITT (PPD-B) reactions of goats infected with *M. bovis* can be bigger than in goats infected with *M. caprae* as well^29^.

Both FP and PCL showed the highest performance parameter estimates with all animals in *M*. *bovis* and *M*. *caprae* groups being clear reactors (100% apparent sensitivity), while no reaction was observed in any of the remaining guinea pigs (100% specificity). In contrast to P22, the mean erythema areas caused by PCL and FP were similar in *M*. *bovis* and *M*. *caprae*-sensitized guinea pigs. In addition, FP reactions in these two groups were similar to or even greater than those caused by PPD-B, which suggests that this antigen would have a comparable sensitivity with a greater specificity.

MKC is of great significance in terms of induction of nonspecific reactions in TB diagnosis because it seems to be cross-reactive not only with PPD, but also with defined antigens based on ESAT-6 or CFP-10^15,17,30^. The isolate from the complex used in this study, *M. persicum*, was obtained from a cow with no recent skin test available but showing gross lesions indistinguishable from typical bovine TB lesions at slaughter. Inoculation of guinea pigs with this isolate did not induce any responses to PCL or FP. Previous research has shown that both *M. kansasii* (former Type I *M. kansasii*) and *M. persicum* (former Type II) harbor ESAT-6 and CFP-10^30,31^. Their ESAT-6 and CFP-10 nucleotide sequences are almost equal (different only in 3 and 2 base positions, respectively) and result in identical amino acid sequences, although expression level is lower in *M. persicum*. Furthermore, a recent report concluded that the type-VII secretion system ESX-1-related EspACD locus, which is required for the secretion and function of EsxA (ESAT-6) and EsxB (CFP-10), is absent from *M. persicum* isolates^32^. Despite this, the effect of increasing the challenge dose of this isolate should be investigated also.

As expected, *M*. *microti* sensitization did not produce visible reactions towards these defined antigens as a result of its genomic deletions^33^. But it was also a low sensitizing power organism (including PPDs) at least using the SD sensitization, with guinea pigs mostly undetectable with confirmatory tests. The low responses to PPD-B in this group might not be in line with expectations. It is not clear if this could be due to differences between the abundance of the antigens present in PPD-B^9^ and those secreted by *M. microti*^33^ (*M. microti* in general or this specific strain) or to a less successful sensitization. These conclusions should be reassessed using higher sensitizing doses and other strains. Notwithstanding, the epidemiologic situation of *M*. *microti* in certain geographic regions does not seem to be irrelevant^34–36^. Thus, a combined use of defined antigens together with PPD-B might help distinguish between *M*. *bovis* or *M*. *caprae* infections and *M*. *microti* infections if they can be suspected.

In conclusion, in light of these and previous reports there is a need to at least complement the standard tuberculins with new defined antigens PCL and FP^7^ or additional antigens or diagnostic interpretation criteria. Further research is necessary to assess the effects of sensitization with more NTM and bacteria other than mycobacteria (e.g. *Corynebacterium* sp. and *Nocardia* sp.), at different sensitizing doses inoculated through different routes, including co-sensitization with non-MTC and MTC bacteria and with a special focus on microorganisms already suspected of causing interferences in the diagnosis of TB, as well as to estimate analytical and epidemiologic performance parameters of defined PCL and FP antigens, all of that considering animal species subjected to routine TB testing (i.e. cattle and goats).

## Methods

### Guinea pigs

Two consecutive experiments were carried out. Thirty-nine and fifteen specific pathogen-free Dunkin Hartley (HsdDhl:DH) female guinea pigs weighing 300–349 g were purchased from Envigo (Envigo, Horst, Netherlands) and used in the first and the second experiments, respectively. Since all were experimental animals, no inclusion/exclusion criteria were set a priori except sex and weight. Animals were randomly distributed (no specific randomization method) in groups of three individuals each (I, II and III) and housed separately in GP-SUITE cage racks (Tecniplast S.p.A., Buguggiate, Varese, Italy) with ad libitum water and food supply at the biosafety level 3 animal facilities in NEIKER. All animals had a two-week adaptation period. Thirteen groups were used in the first experiment, including standard dose (SD) *Nocardia* sp., *Rhodococcus equi*, *M*. *monacense*, *M*. *nonchromogenicum*, *M*. *intracellulare*, *M*. *avium* subsp. *avium*, *M*. *avium* subsp. *hominissuis*, *M*. *avium* subsp. *paratuberculosis*, *M*. *scrofulaceum*, *M*. *persicum*, *M*. *microti* and *M*. *caprae* groups as well as a negative control group. The second experiment comprised five groups that included higher dose (HD) groups of *M*. *monacense*, *M*. *nonchromogenicum*, *M*. *intracellulare* and *M*. *avium* subsp. *paratuberculosis* and an SD group of *M. bovis* (see “Intramuscular inoculation of bacteria” section). The number of animals per group was the minimum. It was determined by the minimum number of individuals needed for a Latin Square design allowing for changing the antigen injection position (6 antigens) between the animals of each group, but considering each flank as an independent experimental unit in order to reduce the number of animals needed (see “Skin test” section for more detail). Each experiment started after a 2-week adaptation period. The order of treatments and measurements and animal/cage location were not considered as potential confounders. Only two investigators performed the intramuscular inoculation of bacteria and were aware of the treatment and control groups allocation (M.V.G. and I.A.S.).

Animal housing and care, as well as all the experimental procedures were carried out in compliance with the European, National and Regional Law and Ethics Committee regulations. The experimental design underwent ethical review and approval by NEIKER’s Animal Care and Use Committee (NEIKER-OEBA-2020-010) and by the competent local authority, the Department of Agriculture of Diputación Foral de Bizkaia (2020/52-BFA).

### Bacteria

The bacterial strains used to inoculate the guinea pigs are listed in Table 4. All NTM were isolated from SITT reactor cattle or cattle with TB-compatible lesions that were negative to MTC in culture. All mycobacteria were grown in Middlebrook 7H9 (M7H9) broth (Becton, Dickinson and Company, Sparks, MD, USA) supplemented with 10% Middlebrook oleic acid-albumin-dextrose-catalase (OADC) enrichment (Becton, Dickinson and Company), 0.2% glycerol and 0.05% Tween 80 (Sigma-Aldrich, Co. Ltd., Haverhill, United Kingdom). *M. avium* subsp. *paratuberculosis* culture broth was further supplemented with 2 mg/l mycobactin J (IDvet, Grabels, France). *Nocardia* sp. and *Rhodococcus equi* isolates were propagated in 5% sheep blood Columbia agar plates (bioMérieux, Marcy-l’Étoile, France) and re-suspended in phosphate-buffered saline containing 0.2% glycerol and 0.05% Tween 80 (PBS-GT). The concentration of bacteria was normalized using the pelleted wet weight method proposed earlier for *M. avium* subsp. *paratuberculosis* as follows^37^. The bacterial cells were harvested by centrifugation at 3000×*g* for 10 min in a pre-weighed cone-bottomed centrifugation tube. The supernatant was decanted and the tubes drained for 10–15 min on sterile absorbent material. The tube was reweighed and the wet weight of the bacterial pellet determined. Bacteria were re-suspended at a final concentration of 2 mg/ml in PBS-GT and stored at − 80 °C until use. The concentration of colony-forming units (CFU) of these stock suspensions was estimated by plating serial dilutions onto agar-solidified M7H9 with 10% OADC and 0.2% glycerol (mycobacteria) or onto 5% sheep blood Columbia agar (non-mycobacteria) the day of inoculation.

**Table 4:** Bacterial isolates used in the study.

Strains	Designation	Isolated from	Laboratory of origin
*Rhodococcus equi*	20Z3743	Cattle with TB-like lesion(s)	ANSES
*Nocardia* sp.	17Z03679	Cattle with TB-like lesion(s)	ANSES
*M. nonchromogenicum*	2416	SITT-reactor cattle	ANSES
*M. monacense*	6306	SITT-reactor cattle	ANSES
*M. intracellulare*	18.00740_001.06	SITT-reactor cattle	NEIKER
*M. avium* subsp. *paratuberculosis*	MAP832	SITT-reactor cattle	NEIKER
*M. avium* subsp. *avium*	18.00616_001.03	SITT-reactor cattle	NEIKER
*M. avium* subsp. *hominissuis*	17.02242_001.02	SITT-reactor cattle	NEIKER
*M. scrofulaceum*	15.05910_001.03	SITT-reactor cattle	NEIKER
*M. persicum* (MKC))	B-00363-18	Cattle with TB-compatible lesion(s)	IRTA-CRESA
*M. microti*	16Z002093 (SB2272)	Badger	ANSES
*M. caprae*	OVIONE (SB0157)	Sheep	IRTA-CRESA
*M. bovis*	2575/08 (SB0339)	Wild boar	NEIKER

### Intramuscular inoculation of bacteria

Bacteria inocula were prepared the same day of inoculation by diluting the frozen 2 mg/ml stocks (− 80 °C) in sterile saline (NaCl 0.9%) solution (B. Braun Medical S.A., Rubí, Barcelona, Spain). The procedure for guinea pig sensitization and skin testing was based on the recommendations of the WOAH for tuberculin potency testing^27^. In the groups of the first experiment and in the second experiment’s *M. bovis* group, animals were inoculated 0.5 ml of saline solution containing a SD (0.0001 mg wet weight corresponding to approximately 10^3^ CFU) of the corresponding microorganism by a deep intramuscular injection made on the medial side of the thigh of the right hind limb. Inoculations with *M*. *nonchromogenicum*, *M*. *monacense*, *M*. *intracellulare* and *M*. *avium* subsp. *paratuberculosis* were repeated in the second experiment using new guinea pig groups and a HD of these bacterial species (0.1 mg wet weight corresponding to approximately 10^6^ CFU). Animals in the negative control group were inoculated with 0.5 ml of the same saline solution used to prepare bacteria inocula.

### Skin test

**Figure 3:**
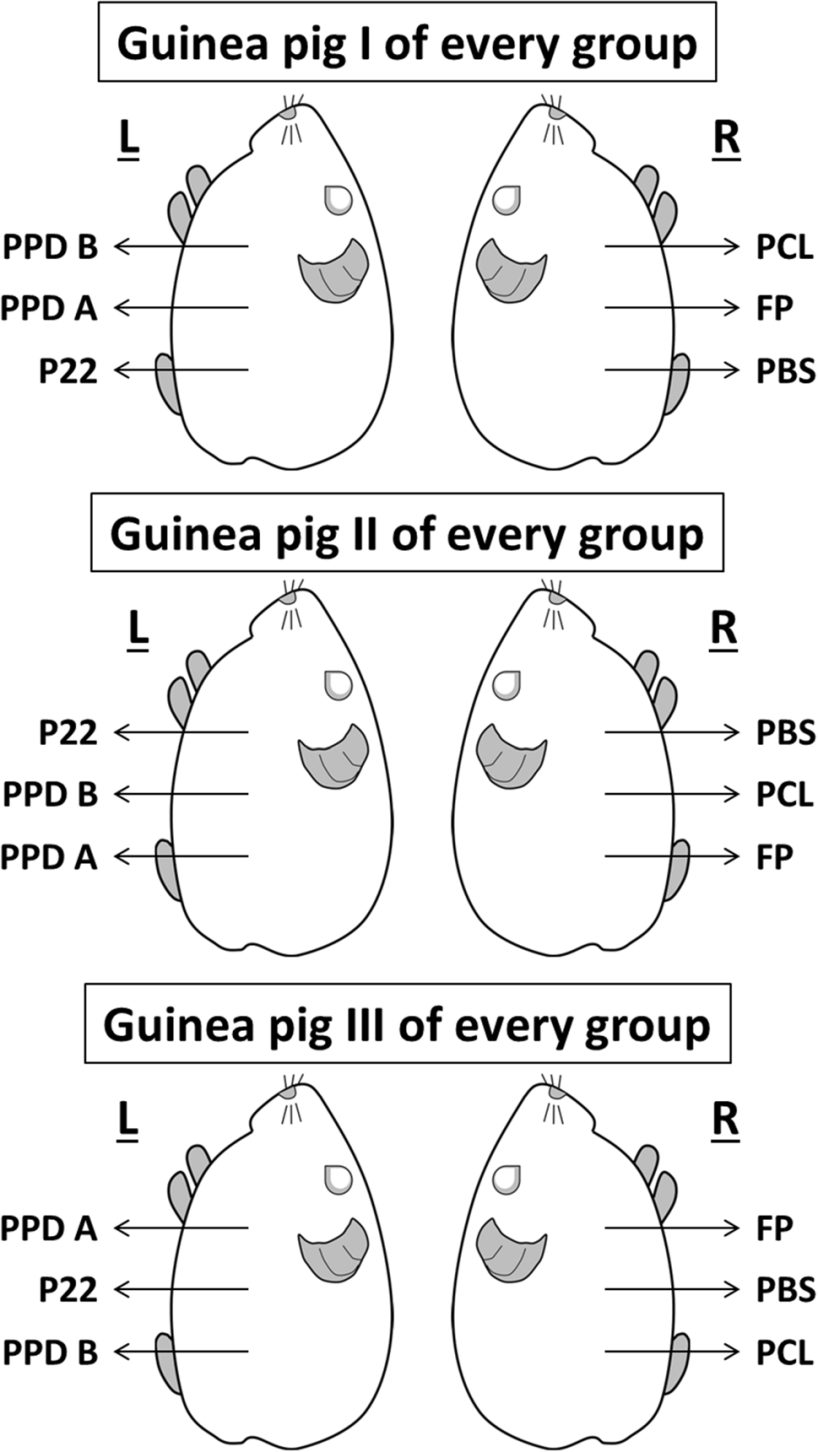
Position of skin test antigens. Site of injection of skin test antigens in guinea pigs according to a Latin square arrangement considering each side of each animal as independent.

Five weeks after inoculation both flanks of guinea pigs were depilated. According to our previous experience and to the literature, the skin test reaction magnitude is not significantly influenced by the location in the flank of the guinea pig or the flank itself (right or left) where an antigen is injected^38^. However, skin test antigens were inoculated in a Latin square design in agreement with WOAH recommendations but considering each flank of each guinea pig as independent experimental units (see Fig. 3). Antigens were diluted in saline solution to deliver the desired dose of each antigen in 0.1 ml intradermal injections using MYJECTOR (29G × 1/2′′) 1 ml insulin syringes (Terumo Europe N. V., Leuven, Belgium). In the left flank, 2 µg of PPD-B (50 IU), 1 µg of PPD-A (50 IU) (CZ Vaccines, Pontevedra, Spain) and 2 µg of P22^9^ were injected in alternate positions. In the right flank, the defined antigens peptide cocktail-long (PCL) (1 µg each peptide) (GenScript Biotech, Piscataway, NJ, USA) covering the sequences of ESAT-6, CFP-10, and Rv3615c^7^ and ESAT-6-CFP-10-Rv3615c fusion protein (FP) (3 µg) (Lionex Ltd., Braunschweig, Germany) as well as a no-antigen negative control (saline solution) were injected. Skin test responses were measured after 24 h and 48 h by the same investigator (J.M.G.; unaware of grouping) and using calipers. The area (mm^2^) of erythema was calculated according to its shape (circumference A = π × r^2^; ellipse A = π × major r × minor r).

### Post-mortem analysis

After skin test response readings, animals underwent deep general anaesthesia with intramuscular xylazine (5 mg/kg; XILAGESIC 2%, Laboratorios Calier SA, Barcelona, Spain) and ketamine (50 mg/kg; Anesketin 100 mg/ml, Dechra Veterinary Products, Barcelona, Spain). Guinea pigs were then euthanized by intracardiac administration of sodium pentobarbital (200 mg/kg; Dolethal 200 mg/ml, Vetoquinol Especialidades Veterinarias, Madrid, Spain). All animals were systematically and thoroughly necropsied.

#### Gross pathology and histopathology

Guinea pigs’ tissues were visually inspected in search of macroscopic pathological changes. Whenever possible, samples from retropharyngeal, parotid, cervical, prescapular, tracheobronchial, mediastinal, axillary, iliac, mesenteric, hepatic, prefemoral, inguinal and popliteal lymph nodes (LNs), as well as from lungs, liver, spleen, kidneys, intestine and muscle tissue from the inoculation injection site were collected for pathological and microbiological analysis. However, sample representation might have not been equal for histopathological and microbiological analysis always. Thus, a sample showing microscopic lesions could have been underrepresented in culture or inversely, a culture positive sample could have been underrepresented in histopathological analysis.

Tissue samples were fixed in 10% buffered formalin and subsequently dehydrated through a graded alcohol series before being embedded in paraffin wax. Sections, 3–5 μm thick, were stained with Carazzi's haematoxylin and eosin (HE) for histopathological studies and with ZN method for acid‐fast bacilli (AFB) detection.

#### Culture

A pool including tissues from the digestive system (pool 1; intestine portion including the ileocecal junction and mesenteric LNs), a pool with tissues from the respiratory tract (pool 2; lungs and associated lymph nodes) and a pool including tissues most related with potential lymphatic or hematogenous circulation of bacteria (pool 3: liver, spleen and retropharyngeal, parotid, cervical, prescapular, axillary, iliac, hepatic, prefemoral, inguinal and popliteal LNs) were cultured. If macroscopic pathological changes were observed in muscle tissue from the site where bacteria were injected, this tissue was also cultured.

Tissues (0.2–2 g) were thoroughly homogenized in 10 ml of sterile distilled water using a GentleMACS™ Dissociator (Miltenyi Biotec, Bergisch Gladbach, Germany). Tissue homogenates from mycobacteria-exposed guinea pigs were decontaminated with the BD BBL™ MycoPrep™ kit (Becton, Dickinson and Company, Sparks, MD, USA) following the instructions of the manufacturer. The resulting pellet was re-suspended in 1 ml sterile distilled water. The suspension was used to inoculate one BBL™ mycobacteria growth indicator tube (MGIT™) supplemented with BACTEC™ MGIT™ growth supplement and PANTA™ antibiotic mixture (Becton, Dickinson and Company), one Coletsos tube (Difco, RPD SL, Sentmenat, Barcelona, Spain) and one tube of Lowenstein-Jensen medium with pyruvate (Difco). Samples from *M*. *avium* subsp. *paratuberculosis*-exposed animals were also cultured in BD Herrold’s Egg Yolk Agar with Mycobactin, amphotericin, nalidixic acid and vancomycin (Becton, Dickinson and Company). Tissue homogenates from guinea pigs exposed to *Nocardia* sp. and *R*. *equi* were seeded onto 5% sheep blood Columbia agar and BD MacConkey agar (Becton, Dickinson and Company) plates. MGIT cultures were introduced in a BACTEC™ MGIT™ 960 System (Becton, Dickinson and Company) and the remaining solid cultures in a standard incubator at 37 °C.

#### PCR identification of isolated mycobacteria

One milliliter of BACTEC positive MGITs was centrifuged at 16,000×*g* for 3 min and the pellets re-suspended in 250 µl of distilled water. Colonies grown on solid media were re-suspended directly in distilled water. Suspensions were transferred to microcentrifuge tubes containing 0.3–0.4 ml zirconia/silica beads (0.1 mm diameter) (BioSpec Products Inc., Bartlesville, OK, USA), inactivated at 90 °C for 20 min and shaken in a TissueLyser II (Qiagen, GmbH, Hilden, Germany) for 10 min at 30 Hz. After centrifugation at 16,000×*g* for 5 min, 5 µl of supernatants was analyzed using a real-time PCR that simultaneously detects the genus *Mycobacterium*, the *M*. *avium* subspecies, the MTC and an internal amplification control^39,40^. DNA from MTC-confirmed isolates was subsequently submitted to standard spoligotyping^41^.

### Statistics

Skin swelling diameter readings were submitted to a first general analysis of variance with the SAS GLM procedure (SAS, Inc. Cary, N. Caroline, USA) for each sensitizing agent (18 levels: *M*. *bovis*, *M*. *caprae*, *M*. *avium* subsp. *avium*, *M*. *avium* subsp. *hominissuis*, *M*. *persicum*, *M*. *microti*, *M*. *avium* subsp. *paratuberculosis* standard and high dose (SD and HD), *M*. *intracellulare* SD and HD, *M*. *monacense* SD and HD, *M*. *nonchromogenicum* SD and HD, *M*. *scrofulaceum*, *Nocardia* sp., *Rhodococcus equi* and blank control), reading time (two levels: 24 and 48 h) and diagnostic reagent (six levels: PPD-B, PPD-A, P22, PCL, FP and PBS) (Table 1). Then a simplified significant model was used for final analysis where factors without significant effect and sensitizing levels without any response were excluded (Tables 1, 2). Time was also excluded once verified that readings decreased by 48 h. Finally, post-hoc PPD-B and P22 responses least square means pairwise comparisons with the Student’s t test Tukey correction for multiple comparisons were applied for the sensitizations with *M. bovis* and *M. caprae* in comparison to the other sensitization groups (Table 2).

### Ethics declaration

The authors confirm that the ethical policies of the journal, as noted on the journal’s author guidelines page, have been adhered to. The study is reported in accordance with ARRIVE guidelines.

## Data Availability

All the data are available in the publication, including the tables and figures.
